# “Bone-SASP” in Skeletal Aging

**DOI:** 10.1007/s00223-023-01100-4

**Published:** 2023-05-31

**Authors:** Ching-Lien Fang, Bin Liu, Mei Wan

**Affiliations:** 1 Department of Orthopaedic Surgery, The Johns Hopkins University School of Medicine, Baltimore, MD 21205 USA; 2 Department of Orthopaedic Surgery, The Johns Hopkins University School of Medicine, Ross Building, Room 209, 720 Rutland Avenue, Baltimore, MD 21205 USA

**Keywords:** Bone-SASP, Cellular senescence, Osteoarthritis, Osteoporosis, Premature aging syndromes, Progeria syndrome, Senescence-associated secretory phenotype (SASP), Skeletal aging

## Abstract

Senescence is a complex cell state characterized by stable cell cycle arrest and a unique secretory pattern known as the senescence-associated secretory phenotype (SASP). The SASP factors, which are heterogeneous and tissue specific, normally include chemokines, cytokines, growth factors, adhesion molecules, and lipid components that can lead to multiple age-associated disorders by eliciting local and systemic consequences. The skeleton is a highly dynamic organ that changes constantly in shape and composition. Senescent cells in bone and bone marrow produce diverse SASP factors that induce alterations of the skeleton through paracrine effects. Herein, we refer to bone cell-associated SASP as “bone-SASP.” In this review, we describe current knowledge of cellular senescence and SASP, focusing on the role of senescent cells in mediating bone pathologies during natural aging and premature aging syndromes. We also summarize the role of cellular senescence and the bone-SASP in glucocorticoids-induced bone damage. In addition, we discuss the role of bone-SASP in the development of osteoarthritis, highlighting the mechanisms by which bone-SASP drives subchondral bone changes in metabolic syndrome-associated osteoarthritis.

## Introduction of Cellular Senescence and the SASP

### Definition and the Stresses Triggering Cellular Senescence

Cellular senescence is a stress response that leads to a stable cessation of the cell cycle, halting the growth of damaged and potentially harmful cells. It is marked by morphological changes, such as flattened cell shape, resistance to apoptosis, activation of DNA damage response (DDR), and a complex and tissue-specific senescence-associated secretory phenotype (SASP), in which senescent cells secrete various factors that can have both beneficial and detrimental effects on neighboring cells and tissues. Senescence can be triggered by various types of stress, such as telomere damage/shortening [1, 2], DNA damage [3–6], reactive oxidative stress (ROS) [7, 8], inflammation [9, 10], mitochondrial dysfunction [11], and oncogene activation [12, 13]. Telomere shortening and DDR may be the most studied mechanisms that induce senescence. Telomere shortening occurs during cell division because of the “end replication” problem [14]. Telomeres are shortened by 50–200 bp with each round of somatic cell division and have been shown to shorten during aging with various human somatic cell types both in vitro and in vivo [15, 16]. DDR is a complex signal transduction pathway that is responsible for sensing and responding to various types of DNA damage. Such responses include DNA lesion repair, transient cell cycle arrest, apoptosis, and senescence. Normally, DDR induces a transient cell cycle arrest, allowing sufficient time for the repair machinery to act on DNA lesions and repair the damage [17]. After repair, the arrested cell exits from the arrest and resumes cell cycle progression [17]. It has been evinced that persistent DDR foci are often associated with telomeres that are exposed to DNA damage, whether it is induced endogenously by oxidative stress or exogenously by genotoxic agents [18–20]. Loss of mitochondrial function is another key contributor to cellular senescence and is also a hallmark of aging [21]. Mitochondria plays an important role in energy production through oxidative phosphorylation, in which they can generate ATP by oxidizing NADH to NAD + [22]. Mitochondrial dysfunction lowers the conversion of NADH to NAD +. Reduced NAD + /NADH ratio and impaired mitochondrial function lead to elevated ROS production, which could further cause cellular damage and DNA mutations for cellular senescence [23–25].

### Senescence-Associated Cell Cycle Arrest

Cell cycle arrest is a common feature of cellular senescence. Senescent and quiescent cells have common molecules that play a role in determining cell cycle arrest. However, these two cell states have distinctive phenotypes at both molecular and morphological levels. Whereas quiescence is a temporary arrest state, in which the cell retains the ability to re-enter cell proliferation, growth arrest in senescence is permanent, making the cell unable to resume proliferation in response to any growth factors or mitogenic stimuli [26–28]. Another characteristic that sets these two states apart is that quiescent growth arrest takes place during the G0 phase [29], whereas senescent cells are halted during the G1/S phase and possibly the G2/S phase [30]. Senescent cells are also distinct from terminal differentiated cells, in which terminal differentiation is a defined developmental program, whereas senescence is a cellular stress response mediated by different pathways [27, 31, 32]. The two main signaling pathways involved in cellular senescence are the p53/p21Cip1 and p16INK4A tumor suppressor pathways. Various stress factors described above trigger the DDR pathway, which in turn activates the p53 and/or the p16INK4A pathways. p16INK4A inactivates Cdk4/6 for the accumulation of phosphorylated pRb, which stops the regulation of E2F transcription factors and drives cell cycle arrest or senescence. These stressors also trigger ATM-Chk2 or ATR-Chk1 pathways and transactivate p53 and p21CIP1, which lead to the inhibition of Cdk4/6 activity and consequent G1 arrest or senescence [33–35].

Senescence is often accompanied by morphological changes, specifically flattening and increasing in size [36, 37]. Cells are large because cell division is blocked by cell cycle arrest; however, macromolecule biosynthesis still occurs and continues to drive cell growth. As a result, senescent cells increase in size without a corresponding increase in DNA content [38]. Another hallmark of senescent cells is their resistance to apoptosis, through the upregulation of senescent cell anti-apoptotic pathways (SCAPs) [39–42]. Based on the above characteristics of senescent cells, several common markers are often used to identify senescent cells in in vitro and in vivo studies, including changed morphology, increased senescence-associated β-galactosidase (SA-βGal), telomere associate foci, senescence-associated distension of satellites, senescence-associated heterochromatin foci, activation of cell cycle inhibitors/tumor suppressors (e.g., p16INK4a, p19INK4d, p21Cip1), and the SASP. Despite these features, it remains a challenge to effectively and comprehensively identify the senescent cells in vivo mainly due to the heterogeneity of the cells. Very recently, Cherry et al. developed an in vivo-derived senescence signature (SenSig) using a fibrosis model in a senescence reporter mouse [43]. Further, using a transfer learning technique to score mouse and human scRNA-seq datasets for concordance with the SenSig, the group identified two senescent cell populations. The SenSig transfer learning approach provides a robust method to identify senescent cells in would healing and other age-related pathologies across tissues and species.

It is worth mentioning that cellular senescence may underlie sex differences in senescence pathologies. Current studies show that female sex is associated with greater susceptibility to DNA damage and more prone to senescence in many experimental models, such as human peripheral blood lymphocytes, peripheral blood mononuclear cells, and others [44]. For instance, DSBR via NHEJ declines with age in women, but not in men in peripheral blood lymphocytes [45]; female cells undergo senescence, while male cells undergo apoptosis following UV irradiation in rat vascular smooth muscle cells [46]. Consistent with these findings, a recent study showed that the neurons and glial cells of mice that underwent repeated mild traumatic brain injury acquired a senescent signature, with female mice having higher levels of DNA damage, lower levels of the senescence protein p16, and lower levels of the cyclic GMP–AMP synthase stimulator of interferon gene (cGAS-STING) signaling proteins compared with their male counterparts [47]. Sex differences in cellular senescence may underlie sex-specific disease outcomes [48].

### Senescence-Associated Secretory Phenotype (SASP)

A fundamental feature of cellular senescence is the secretion of inflammatory transcriptome, also known as SASP. Early studies of SASP documented many factors secreted from senescent cells, most of which are pro-inflammatory proteins [34, 38]. Among them, interleukin (IL)-1, IL-6, IL-8, chemokine ligands, monocyte chemotactic protein (MCP)-1, MCP-2, matrix metalloproteinase (MMP)-1, MMP-3, growth regulated oncogene (GRO)-alpha, GRO-beta, GRO-gamma, and many insulin-like growth factor-binding proteins are the highly induced and secreted factors [9, 49–54]. The composition of SASP has now become better understood. Recent reports have shown that SASP is also composed of various proteins and non-protein signaling molecules, such as hemostatic factors, ceramides, bradykinins, extracellular matrix components, damage-associated molecular patterns, ROS, and prostaglandin [55–58]. Other SASP components include vesicles, exosomes, various microRNAs and noncoding RNAs, certain fragments of DNA, other nucleotides, protein aggregates, and lipid components [59–62]. The senescent cells and chronic inflammation induced by the SASP contribute to the pathogenesis of many age-related diseases, such as atherosclerosis [63], neurodegenerative diseases [64], frailty [65], and osteoarthritis (OA). SASP is also known to contribute to frailty and several age-associated bone disorders, such as osteoporosis and OA (details are summarized in Section II).

**Fig. 1:**
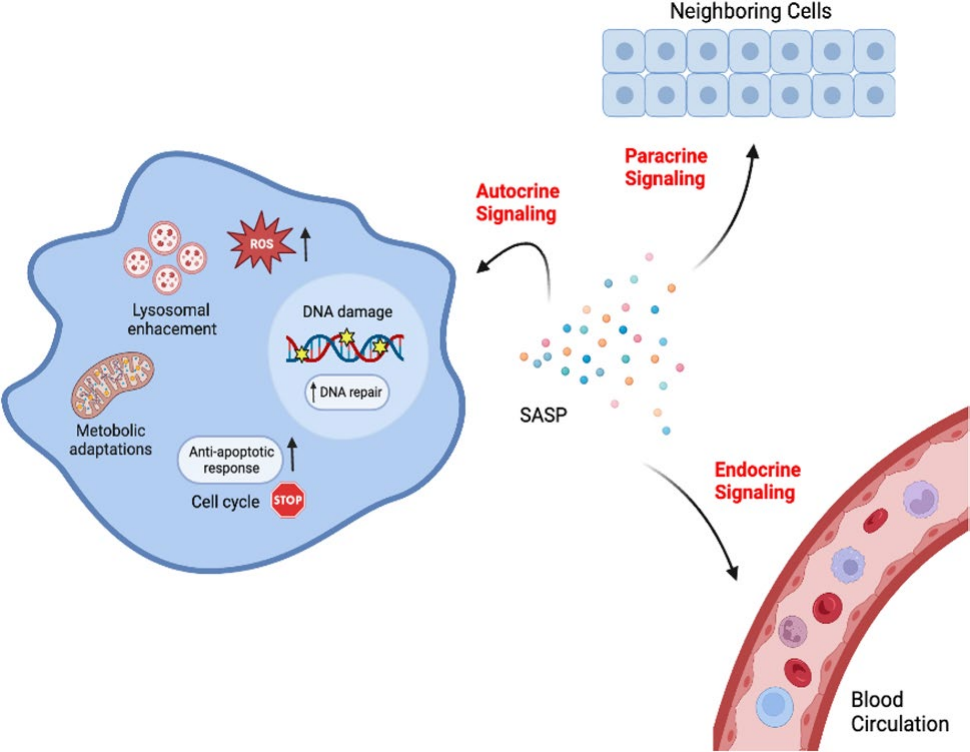
Different types of action of SASP. Stress stimuli can trigger normal cells to enter senescence-associated cell cycle arrest, which is characterized by enlarged and flattened cell shape, lysosomal enhancement, metabolic adaptations, elevated anti-apoptotic response, increasing ROS, and the secretion of SASP. The SASP can have an autocrine effect to reinforce the senescent phenotype and also function in a paracine manner to trigger cellular senescence or regulate the activities of neighboring cells. Endocrine effects of SASP on remote tissues/organs have also been proposed

The SASP composition and strength are highly dynamic, depending majorly on the cell types, senescence inducers, and durations of senescence. A study using proteomic analysis identified heterogeneous SASP profiles with distinct human primary cell types, fibroblasts, and epithelial cells, triggered by different senescent inducers, including genotoxic stress–induced, oncogene-induced, and treatment-induced senescence [55]. Each profile is composed of hundreds of largely distinct proteins but also comprises a core of SASP components commonly elevated in all SASPs: chemokine C-X-C motif ligand, MMP-1, and stanniocalcin (STC)-1. Notably, some SASPs overlap with pro-aging markers in human plasma, including growth/differentiation factor-15 (GDF-15), STC1, and serine protease inhibitors [55]. SASP composition and strength are also regulated temporally. In oncogene-induced senescence, fluctuations in NOTCH1 level can switch an early TGF-β-rich immunosuppressive secretome to a pro-inflammatory SASP [66]. Moreover, during late senescence, the depression of LINE-1 retrotransposable elements serves as a switch for the activation of type-I interferon expression, which is a phenotype for late senescence [67]. Early secretion of the SASP factor PDGF-AA by senescent cells accelerates wound healing and promotes myofibroblast differentiation [68]. However, senescent cells at the wound site also subsequently chemo-attract their own immune-mediated clearance that could delay wound healing, suggesting a temporal switch between wound repair and inflammatory recruitment of immune cells. The dynamic nature and sometimes contradicting effects of the SASP help explain the diverse biological functions associated with senescence. Some components of the SASP can propagate or reinforce the senescent phenotype through autocrine or paracrine mechanisms (Fig. 1), leading to further secretion and amplification of the SASP [69, 70]. In an autocrine manner, SASP reinforces cell autonomous mechanisms, such as cell cycle arrest to the senescent cells themselves. SASP also signals in a paracrine fashion with multiple effects on neighboring cells, such as triggering cellular senescence of surrounding cells, also known as paracrine senescence [71]. The importance of the SASP in eliminating senescent cells through the immune system was emphasized by the discovery that Bromodomain-containing protein 4 (BRD4), an epigenetic regulator that controls the enhancer and super-enhancer architecture of SASP genes and governs the SASP’s capacity to facilitate the immune clearance of senescent cells [22]. Immune-mediated clearance of senescent cells suppresses tumor initiation [72], contributes to tumor regression [73], and is essential during embryonic development [74, 75] and even for the termination of a senescence-associated inflammatory response, preventing chronic inflammation [71].

## Cellular Senescence and Bone-SASP in Natural Aging and Premature Aging Syndromes

All organ systems change with age, resulting in compromise or even loss of function of organs. The skeleton, our body’s central framework, serves many important functions, including body support, facilitation of movement, protection of internal organs, storage of minerals, hematopoiesis, and production of important factors/hormones with diverse effects both locally and systemically. With aging, these functions become altered or impaired. One common condition caused by skeletal aging is bone loss that results in osteoporosis, a common age-associated disorder characterized by low bone mass and bone tissue micro-architectural deterioration with consequent increase in fracture risk. Research during the past decade has clearly demonstrated the presence of senescent cells and the corresponding SASP in the skeleton during aging. The first comprehensive characterization of senescent cells and the SASP in the mouse and human bone/bone marrow microenvironment was conducted by Farr et al. The authors revealed that *p16*^*Ink4a*^ expression is upregulated in multiple bone/bone marrow cell types, including B cells, T cells, myeloid cells, osteoprogenitors, osteoblasts, and osteocytes [76]. Moreover, myeloid cells and osteocytes were identified as the major cell types that have marked upregulation of SASP factors [76]. Piemontese et al. consistently demonstrated that osteocytes and osteoblast progenitors developed markers of cellular senescence with aging [77]. Importantly, Farr et al. provided convincing evidence to support the causal role of senescent bone cells in mediating age-associated bone loss using pharmacological and genetic approaches to eliminate the senescent cells [78]. Recent work by Ambrosi et al. uncovered that aged skeletal stem cells exhibited bone-SASP-like features with high expression levels of pro-inflammatory and pro-resorptive cytokines, contributing to the transformation of the bone marrow niche [79]. As a result, the aged skeletal stem cells promoted osteoclastic activity and myeloid skewing by haematopoietic stem and progenitor cells. It is worth noting that mechanisms mediating cellular senescence may vary depending on different stimuli, even in the same tissue context. For example, while clearance of p16Ink4a‐expressing senescent cells prevents age‐related bone loss [78], clearance of *p21*^+^ but not *p16*^+^ senescent cells prevents radiation‐induced osteoporosis [80]. Recently, Saul et al. validated these findings at a single-cell level by generating a gene set named SenMayo, consisting of 125 previously identified senescence/SASP-associated factors [81]. Importantly, the group demonstrated that clearance of senescent cells in mice and humans resulted in significant reductions of SenMayo, confirming that this is a specific senescence gene set rather than just an “aging” gene set. They further showed that the SenMayo dataset is applicable across tissues and species and performed better than six existing senescence/SASP gene panels. By applying SenMayo to scRNA-seq data, the group identified bone marrow monocytes/macrophages and mesenchymal cells expressing high levels of senescence/SASP markers in the context of aging. Given that identification of the SASP at the single-cell level has been challenging because of the heterogeneity and tissue-specific nature of the SASP, SenMayo provides a standardized gene set that is useful for identifying and characterizing senescent cells and the associated SASP in bone/bone marrow during aging, in different pathological conditions, as well as for evaluating the efficiency of senolytic therapies.

In addition to “natural” or “healthy” aging, the involvement of cellular senescence has also been investigated in progeroid syndromes, which involve premature organ-specific and/or whole-body aging [82–86]. These human progeroid diseases, such as Hutchinson-Gilford progeria syndrome (HGPS), Werner syndrome (WS), Bloom syndrome, Cockayne syndrome, Seckel syndrome, trichothiodystrophy, and xeroderma pigmentosum, provide a unique window into the pathology of natural aging. These progeroid syndromes are rare congenital/genetic disorders that recapitulate some pathological features of normal aging in an accelerated manner and thus provide potential insights into the natural aging process. Most human progeroid syndromes are caused by either defects in the nuclear lamina or deficiencies in the DNA repair machineries. Interestingly, most progeroid syndromes are characterized by skeletal abnormalities, such as low bone density and osteoporosis. Currently, numerous mouse models of premature aging have been established [87, 88], and these models recapitulate phenotypes of musculoskeletal age-related decline observed in humans. The earliest study identifying accumulated senescent cells and the causal role of cellular senescence in age-associated conditions came from a study using BubR1^H/H^ mice, which have a markedly shortened lifespan, age-associated phenotypes in almost every organ system examined, and severe kyphosis [89]. Removal of senescent cells using the INK-ATTAC mice, in which the p16INK4a-positive senescent cells can be eliminated, delayed the onset of age-related phenotypes in multiple tissues of progeroid mice. Despite efficient repair, DNA damage inevitably accumulates with time, affecting proper cell function and viability, thereby driving systemic aging. Ercc1^−/Δ^ mice are well-characterized DNA repair mutants that exhibit widespread premature aging across many tissues within a lifespan of 4–6 months [90, 91]. ERCC1 is an endonuclease involved in DNA repair pathways. As a result of its mutation, a broad variety of DNA lesions accumulate more rapidly in these mice, causing genomic instability, functional decline, and premature aging. Ercc1^−/Δ^ mice develop a low bone mass phenotype at a young age, faithfully recapitulating the premature aging phenotype of human XFE progeria [92]. Bone marrow stromal cells from Ercc1^−/Δ^ mice exhibited an increase in cellular senescence marker p16INK4a, DNA damage marker γH2AX, and SASP factors, including IL-6, TNFα, RANKL, and OPG [93]. Recently, it was reported that Ercc1^−/Δ^ mice also showed a spinal disc aging phenotype, including loss of disc height and degenerative structural changes in their vertebral bodies similar to those observed in old rodents [94]. It would be interesting to identify the cell types that undergo cellular senescence in the disc and to investigate the key SASP factors mediating the disc and spine-aging phenotype in this premature aging mouse model.

Among the progeroid syndromes, HGPS has been widely studied because patients show a broad range of accelerated aging features. Particularly, skeletal abnormality is one of the extreme phenotypes. HGPS involves lethal premature aging that is caused by mutations in the *LMNA*, a prelamin A protein encoding gene. Prelamin A is the C terminally farnesylated precursor of the nuclear scaffold protein lamin A. Prelamin A is cleaved by the zinc metalloprotease STE24 (ZMPSTE24) shortly after synthesis. In HGPS, genetic mutations in the *LMNA* or *ZMPSTE24* gene lead to defective processing of prelamin A, resulting in premature aging syndromes [82, 95–100]. Lamin A has been implicated in numerous fundamental functions, including maintaining the structural integrity of the nucleus, providing an organizing platform for transcription factors, and regulating mechanical properties of the nucleus [101–104]. Defective laminar organization causes deformed nuclear architecture, leading to loss of genomic integrity and telomere attrition. Of the many different HGPS animal models, mice deficient in Zmpste24 exhibit severe age-associated skeletal deficits, such as growth retardation, kyphosis, low bone mass, and spontaneous bone fracture [105, 106]. Lamin A null mice have reduced trabecular and cortical bone at a young age with fewer osteoclasts and osteoblasts [107]. Lmna^G609G/G609G^ homozygous mice exhibit joint immobility, vertebra and skull deformities, decreased tibial bone mineral density, decreased cortical thickness, and increased porosity [108]. Furthermore, it was reported that the accumulation of prelamin A isoforms at the nuclear lamina triggers an ATM- and NF-κB essential modulator (NEMO)-dependent signaling pathway that leads to NF-κB activation and a SASP (i.e., secretion of high levels of pro-inflammatory cytokines) in both Zmpste24^−/−^ and Lmna^G609G/G609G^ mice [109]. Inhibiting IKK/NF-κB activation in Zmpste24^−/−^ mice reduced markers of cellular senescence and SASP and improved multiple parameters of aging [110]. Recently, Wang et al. generated an *Lmna*^*L648R/L648R*^ mouse line, which is a new progeria mouse model [111]. *Lmna*^*L648R/L648R*^ mice have far less severe aging phenotypes, such as cardiovascular deficits, than that of Zmpste24^−/−^ mice. However, similar to Zmpste24^−/−^ mice, *Lmna*^*L648R/L648R*^ mice have apparent skeletal defects, including decreased vertebral bone density, as well as cranial, mandibular, and dental defects. One of the most common symptoms of progeroid laminopathy is accelerated cellular senescence or aging. Fibroblasts from HGPS patients exhibit features of cellular senescence, such as DNA damage, telomere shortening, disrupted nuclear morphology, and loss of peripheral heterochromatin [112]. Particularly, progerin, excessive accumulation of prelamin A, and downregulation of ZMPSTE24 induce premature senescence in mesenchymal stem cells (MSCs) [113, 114]. In addition, MSCs with both progerin overexpression and ZMPSTE24 depletion have a SASP phenotype, which is mediated by GATA4 [114].

Werner syndrome (WS) is another premature aging disorder with an evident skeletal aging phenotype. WS is caused by loss of *WRN*, the gene encoding an enzyme involved in DNA repair and telomere maintenance. The premature aging phenotypes of WS include short lifespan, early-onset atherosclerosis, cataracts, osteoporosis, type II diabetes mellitus, and an elevated incidence of soft tissue sarcoma [115]. Osteoporosis has been observed in approximately 41% of patients with WS, with bone loss in the femur more severe than that in the lumbar spine. It is postulated that osteoporosis occurs because bone formation is inhibited while bone resorption is normal in WS [116]. A WS mouse model, in which both Wrn and telomerase are deleted, has a bone loss phenotype that is associated with reduced numbers of MSCs and increased replicative senescence of marrow progenitors [117, 118]. As early as 1981, it was found that primary skin fibroblasts from patients with WS undergo early replicative senescence [119]. Moreover, MSCs differentiated from WS iPSCs have a premature senescence phenotype, including epigenetic and chromosomal structure alteration and premature loss of proliferative potential [120]. Zhang et al. found that p21Waf1/Cip1 and p16Ink4a have distinct functions in modulating aging phenotypes of WS [121]. Particularly, p21 loss in WS activated severe DDR. Conversely, p16 deficiency attenuated telomere attrition without causing severe DDR. Senescence is also linked to other hallmarks of aging, such as telomere attrition and mitochondrial dysfunction. These findings demonstrate that deficits in DNA repair, telomere shortening, and epigenetic alterations caused by *WRN* loss promote premature cellular senescence [122]. Tian et al. discovered that, similar to MSCs and the bone aging phenotype, *WRN* deficiency results in the inhibition of bone growth and short stature in vivo [123]. They further found that loss of *WRN* causes chondrocyte senescence characterized by increased SA-βGal^+^ cells and upregulated p53 and p16INK4a mRNA expression and that overexpression of *SHOX*, a direct target of *WRN*, prevents the senescence phenotype in a zebrafish model. These findings highlight the potential involvement of *WRN* deficiency-induced chondrocyte senescence in the regulation of growth plate chondrocytes and bone growth.

Studying age-associated skeletal decline in progeria models has advantages because of the benefits of a short lifespan. These studies have led to the identification of important molecular pathways that impinge on the skeletal aging process. However, much attention should be paid to the interpretation of the outcomes from the progeroid study. Currently, the extent to which progerias resemble natural aging is still debated, given that studying progeria does not address all of the common problems during natural aging. Moreover, some progeria models may show certain characteristics of natural aging but lack others. Fortunately, many premature aging syndromes and progeria models exhibit skeletal abnormalities, such as low bone mass and osteoporosis. Therefore, combined use of the progeroid models and skeletal tissue-specific genetic models may provide more accurate understanding of the mechanisms that drive skeletal aging. Of note, studies using unbiased proteomics and RNA-sequencing approaches to identify the senescent cells and the SASP factors in these progeroid models are still lacking. The application of the SenSig and SenMayo dataset in combination with RNA-sequencing data will be important to fulfill the purpose.

## Cellular Senescence and Bone-SASP in Glucocorticoid-Induced Bone Damage

Although senescent cells are typically associated with aging, evidence suggests that they have important functions in regulating embryonic skeletal development and postnatal bone growth, as recently reviewed in detail [124]. Childhood and adolescence, characterized by rapid physical growth and bone development, are crucial periods for bone health. Our group demonstrated that cellular senescence also plays a role in childhood bone growth-associated bone mass acquisition. We identified a programmed cellular senescence at the metaphysis of long bone during late puberty, when bone growth slows or stops [125]. The senescent cells, characterized by the presence of SA-βGal, loss of nestin, and upregulation of p16INK4a, were primarily mesenchymal progenitor cells. Growth hormone or parathyroid hormone are positive regulators of bone growth/acquisition, as the receptors of these factors/hormones are expressed in metaphysis of long bone [126, 127]. We found that these bone growth-promoting factors inhibited cellular senescence, whereas glucocorticoid treatment exacerbated senescence. Thus, cellular senescence in this bone region and during this period is negatively associated with skeletal growth and bone accrual and may serve as an important signature for the transition from rapid to slow growth in long bone. Further, this programmed cellular senescence is mediated by Ezh2-H3K27me3 [125], suggesting that senescence at the metaphysis is tightly regulated by epigenetic mechanisms. Our findings further suggest that defining the role of cellular senescence in pathological conditions during childhood and adolescence is important because maintaining bone homeostasis during this period helps prevent osteoporosis and reduce fracture risk. Glucocorticoid-induced osteoporosis (GIO) is the most common cause of secondary pediatric osteoporosis. Glucocorticoids are routinely prescribed to treat serious childhood illnesses, including leukemia and other cancers, systemic inflammatory or autoimmune disorders, and neuromuscular disorders, such as Duchenne muscular dystrophy [128], as well as after organ transplantation. Systemic glucocorticoid treatment leads to decreased peak bone mass, architectural deterioration, and increased fracture risk [129, 130]. The pathogenic mechanisms underlying GIO remain incompletely understood, and effective medications to treat childhood GIO are lacking. We uncovered a new mechanism for the deleterious effects of glucocorticoids on the growing skeleton in mice [131]. Cellular senescence occurs in the metaphysis of healthy long bones during the late pubertal period, but glucocorticoid treatment induces a much earlier (prepubertal) cellular senescence in the metaphysis of young mice. We further identified that vascular endothelial cells in type H vessels, which are highly proliferative, and osteogenesis-coupled vessels in the metaphysis, are a primary cell type that becomes senescent in response to glucocorticoids. As a result, angiogenesis and coupled osteogenesis diminish in this region. Moreover, we uncovered the molecular basis for glucocorticoid-induced bone vascular senescence. We found that in healthy growing long bone, an angiogenesis factor angiogenin (ANG) secreted from metaphyseal osteoclasts is essential to maintain the proliferation of the closely associated blood vessels through ANG/PLXNB2-rRNA transcription signaling [131]. Glucocorticoid treatment inhibits ANG production through suppression of osteoclast formation in metaphysis, leading to senescence of blood vessels and the resultant impaired angiogenesis and osteogenesis. Future identification of the SASP factors produced by the senescent bone blood vessels would be important to understand the role of bone-SASP in glucocorticoid-induced childhood bone loss.

**Fig. 2:**
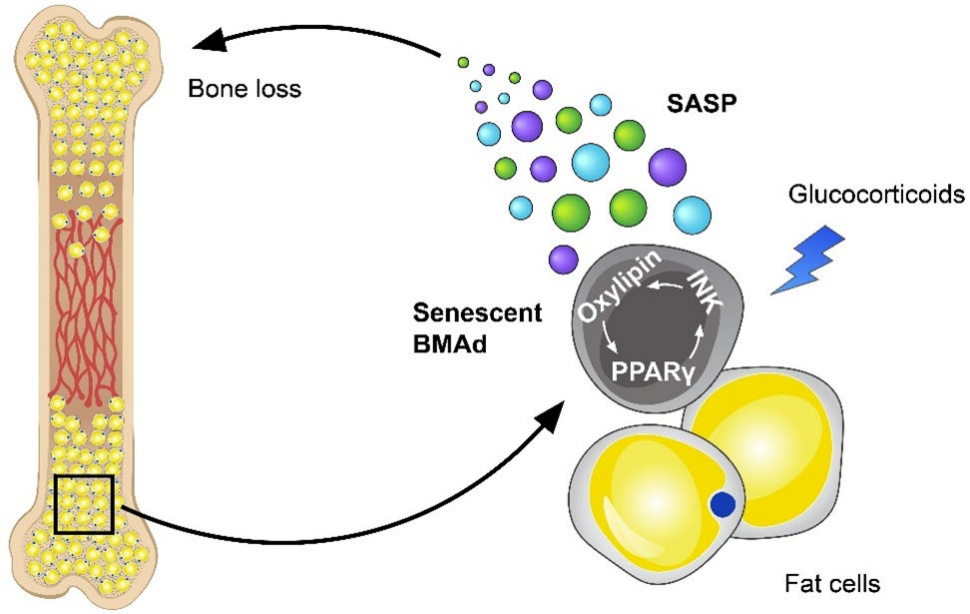
Involvement of senescent BMAds and the SASP in glucocorticoid-induced bone loss. Glucocorticoid treatment induces primary senescence of BMAds through a positive interacting feedback loop of 15d-PGJ2-PPARγ-INK signaling. The senescent BMAds spread senescence to other bone and bone marrow cells, leading to an accumulation of senescent cells for bone impairment

In adults, glucocorticoids are also essential medications because of their powerful anti-inflammatory and anti-allergic effects. During the COVID-19 pandemic, glucocorticoids were recommended by the World Health Organization as one of the preferred medications for severe cases because they can substantially reduce the mortality rate of critically ill patients. Long-term use of glucocorticoids can cause severe adverse effects on the skeleton, such as osteoporosis and bone necrosis. We recently found that glucocorticoid treatment in adult mice induces primary senescence of bone marrow adipocyte (BMAd) lineage cells, which spread senescence to the neighboring bone and bone marrow cells by secreting SASP factors, leading to senescent cell accumulation in the local microenvironment for bone deterioration [132]. In addition to the in situ detection of various cellular senescence markers in bone/bone marrow, we conducted RNA-sequencing and found close to 400 aging/senescence-related genes in the glucocorticoid-treated BMAd lineage cells relative to vehicle-treated cells. We also found a typical SASP expression profile in the dexamethasone (DEX)-treated *vs*. vehicle-treated adipocytes/preadipocytes, compared with the “SASP Atlas,” which is a proteomic database of SASP factors generated by Basisty et al. Many increased genes in the glucocorticoid- vs. vehicle-treated cells are also in the bone-SASP genes in the SenMayo dataset [81]. We further identified a positive feedback loop of 15d-PGJ2-PPARγ-INK signaling that initiates and maintains the senescence of the BMAd lineage cells (Fig. 2). Glucocorticoid treatment increases the synthesis of oxylipins, such as 15d-PGJ2, in BMAds to positively regulate the activity of PPARγ, which stimulates the expression of INK family encoding genes, key cellular senescence effectors. PPARγ activation also promotes oxylipin synthesis in BMAds [132]. The finding suggests that a subtle alteration in this signaling circuit can be amplified, resulting in rapid cellular senescence of BMAds. Furthermore, we evaluated whether the senescent BMAds play a causal role in glucocorticoid-induced bone deficits. It is technically difficult to examine the specific effects of BMAds because adipocytes in the bone marrow and other parts of the body often share the same markers. To address this, we used a bone marrow transplantation approach, in which senescent BMAds from glucocorticoid-stimulated mice were isolated and transplanted into the femoral bone marrow cavity of untreated healthy mice. Using this method, we were able to demonstrate that targeting senescent BMAds attenuates glucocorticoid-induced bone loss. The mechanisms by which excessive glucocorticoids induce bone deterioration have been extensively researched over the past few decades. Glucocorticoids have direct and indirect effects on bone/bone marrow cells, such as osteoblast and osteoclast lineage cells. Our finding of the detrimental effects of senescent BMAds on the bone marrow microenvironment through SASP provides a new clue for the pathogenesis of glucocorticoid-induced bone deterioration [133, 134].

## Bone-SASP in osteoarthritis (OA) Development

Another common effect of aging and senescence on the skeleton is the development of OA, the most prevalent chronic joint disease that affects nearly 250 million people worldwide [135]. OA primarily affects weight-bearing joints and is characterized by progressive articular cartilage degeneration and entire joint dysfunction [136]. The major symptoms of OA are pain and reduced/loss of mobility, imposing substantial mental and physical burdens on the affected individuals and a considerable economic burden on society. Currently, pharmacological treatments mostly aim to relieve the OA symptoms associated with inflammation and pain. To date, no pharmacological agents have been approved by regulatory authorities for disease modification in OA, and ongoing studies are investigating the potential for developing disease-modifying OA drugs [137, 138].

During the past decade, there have been intensive studies of the contribution of chondrocyte senescence to the development of OA. Joen et al. performed the first systemic characterization on senescent cells in joint tissue and examined the causal role of cellular senescence in OA progression using post-traumatic OA (PTOA) animal models [139]. The group identified senescent chondrocytes in cartilage isolated from OA patients and in mice after anterior cruciate ligament transection, which is a PTOA mouse model. Selective elimination of the senescent cells using both genetic and pharmacological approaches attenuated the development of PTOA, reduced pain, and increased cartilage development. The causal role of chondrocyte senescence in PTOA progression has been further confirmed by later studies and was recently reviewed in detail [140–142]. Emerging evidence suggests that senescent cells, via SASP, contribute to an inflammatory state and microenvironmental changes in joint tissue. The SASP factors produced by senescent joint cells include pro-inflammatory cytokines IL-1, IL-6, IL-8, TNFα, chemokines (C–C motif ligand 2, CCL2, CCL4), proteases (MMP-1, 3, 12, 13, and ADAMTS), growth factors, small-molecule metabolites [140, 143, 144], and microRNAs. The SASP factors propagate senescence through paracrine and autocrine mechanisms, further promoting OA progression [145]. Recent accumulating evidence demonstrated that cellular senescence increases the secretion of extracellular vesicles, which can transport proteins and microRNAs that are key components of SASP [146], suggesting that extracellular vesicles may be key mediators in the rapid spreading of senescence in the local joint environment. Based on these studies, senolytic and senomorphic drugs, which can kill the senescent cells and inhibit the SASP, respectively, have been tested for their potential therapeutic effect in OA [140, 147, 148].

Joint cartilage and subchondral bone act in concert as one functional unit [149, 150]. It has been observed clinically that changes in the subchondral bone microarchitecture precede articular cartilage damage in OA [151, 152]. Particularly, aberrant subchondral bone angiogenesis with resultant invasion of vasculature into the osteochondral junction is a hallmark of human OA [153]. In OA mice, accumulating evidence suggests that neo-vessel formation in subchondral bone is characterized by the development of osteogenesis-coupled CD31^hi^Emcn^hi^-type H vessels [154–157]. However, little is known of the cellular and molecular mechanisms of the development of subchondral bone angiogenesis during OA progression. Our group recently found that senescent preosteoclasts secrete much higher level of angiogenesis factor PDGF-BB, which is essential for the development of pathological subchondral bone angiogenesis before the development of OA and during its early stage [158]. We observed a simultaneous increase in type H vessels and osteogenesis in an OA mouse model of destabilization of the medial meniscus. Subchondral preosteoclasts secreted excessive amounts of PDGF-BB in response to traumatic joint injury and increased PDGF-BB activates PDGFR-β signaling in bone/bone marrow vascular cells and pericytes in a paracrine manner for aberrant neo-vessel formation [158]. In that study, we also generated conditional *Pdgfb* transgenic mice (Pdgfb^cTG^) and conditional *Pdgfb* knockout mice (Pdgfb^cKO^), in which PDGF-BB is overexpressed or deleted in TRAP^+^ cells, respectively. Our data show that preosteoclast-derived PDGF-BB is both sufficient and required for pathological subchondral bone angiogenesis and resultant joint degeneration. Particularly, young Pdgfb^cTG^ mice have aberrant subchondral bone angiogenesis with a progressive invasion of new vessels into the joint calcified cartilage, as well as an increase in subchondral bone osteogenesis [158]. The joint phenotype of young Pdgfb^cTG^ mice is quite compelling, because the mice spontaneously develop dramatic subchondral bone alteration during the earlier stage and cartilage degeneration during the later stage. Therefore, Pdgfb^cTG^ mice can serve as a useful spontaneous OA mouse model to enable the study of pathogenic mechanisms and drug treatment.

**Fig. 3:**
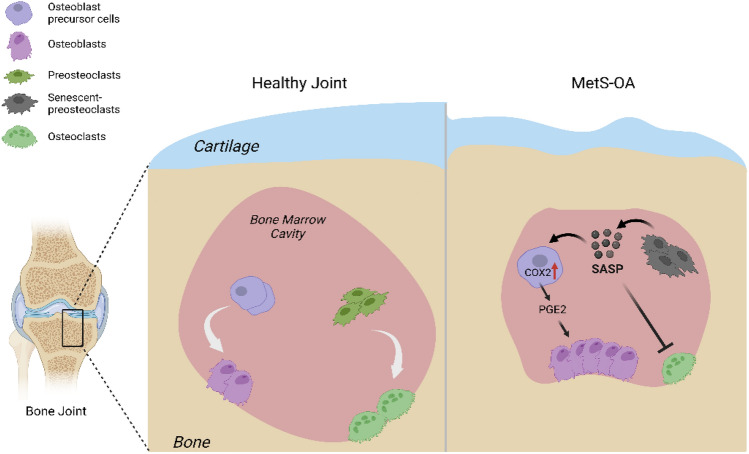
Involvement of preosteoclast-secreted SASP factors in MetS-OA. In normal physiological conditions, the balanced osteoblast and osteoclast differentiation maintain subchondral bone homeostasis and normal subchondral microarchitecture. Under MetS, preosteoclasts in subchondral bone marrow undergo cellular senescence and secrete SASP factors. The SASP acts on both osteoclast precursors to suppress osteoclast differentiation and osteoblast precursors to activate COX2-PGE2 signaling to promote osteoblast differentiation for bone formation, leading to rapid subchondral plate and trabecular bone thickening

OA is a heterogeneous disease with multifactorial causes, various clinical features, and different responses to treatments. Although PTOA is the most studied OA phenotype because of well-established PTOA animal models, non-traumatic OA, especially age- and metabolic syndrome-associated OA (MetS-OA) is more prevalent according to epidemiologic and prospective clinical studies [159–163]. Particularly, metabolic OA is now considered a subtype of OA defined by the presence of individual MetS components or MetS as a whole [159]. Despite these facts, there are limited studies on the involvement of cellular senescence in the pathogenesis of MetS-OA. We recently conducted both human and animal studies that revealed the critical role of cellular senescence in subchondral bone and bone-SASP in driving the progression of Met-OA [164]. In this study, human Osteoarthritis Initiative datasets were analyzed to investigate the subchondral bone features of MetS-OA participants on MRI. Moreover, the joint phenotype of two MetS mouse models, HFD-challenged mice and STR/Ort mice, a well-recognized model that develops spontaneous OA very similar to the human disease, were also assessed. The results show that humans and mice with MetS-OA have a subchondral bone phenotype distinct from that of PTOA and have a greater likelihood of developing OA-related subchondral bone damage. In mice with early-stage PTOA, osteoclast number and activity are increased, with a high turnover rate in subchondral bone [165–167]. However, rapid thickening of subchondral bone plate and trabecular bone occurs in HFD-challenged mice and STR/ort mice [164]. These subchondral alterations appear much earlier than cartilage degradation. Intriguingly, we found that unlike the accumulated senescent cells in cartilage and synovium in PTOA [139, 168], increased senescent cells and the SASP are primarily located in subchondral bone in MetS-OA mice [164](Fig. 3). We identified that many of the senescent cells were RANK^+^TRAP^+^ preosteoclasts in bone marrow. These senescent cells exhibited a unique bone-SASP, containing the canonical SASP factors identified in SenMayo dataset [81], such as IL-1β, IL-6, and VEGF. Other factors secreted by senescent preosteoclasts in subchondral bone of HFD-challenged mice include OPN, Lipocalin-2, Resistin, Cystatin C, IL-33, CCN4, MPO, and PDGF-BB. These factors, however, are not canonical SASP factors based on the SenMayo dataset. Interestingly, most of these factors have been shown to be COX2 gene-activating factors [169–175] and osteoclastogenesis-regulating factors [176–180], suggesting that senescent preosteoclasts acquire a unique SASP that may exert paracrine effects on nearby cells in the subchondral environment. Indeed, our work shows that preosteoclast SASP activates COX2-PGE2 signaling in osteoblast precursors for osteoblast differentiation and inhibited osteoclast differentiation, contributing to rapid subchondral plate and trabecular thickening (Fig. 3). The exact roles of these newly identified preosteoclast-secreted factors in subchondral bone alteration and the progression of MetS-OA remain to be validated.

## Senolytic and Senomorphic Therapy

Senotherapeutic approaches, which are treatments designed to clear or neutralize the effects of senescent cells, have been implicated and evaluated in several models of aging as novel therapeutics [181, 182]. Senotherapeutics can be classified into two main categories: senolytics, which selectively eliminate senescent cells, and senomorphics, which modulate the behavior of senescent cells by regulating the secretion of SASP and help alleviate age-associated chronic diseases. The Kirkland group conducted the first study of its kind [183] and discovered that the combination of dasatinib (D) and quercetin (Q) can effectively eliminate senescent cells and decrease the levels of various proteins. This combination of drugs reduced the burden of senescent cells in both chronologically aged mice and in progeroid Ercc1^−/Δ^ transgenic mice. Moreover, the use of D + Q extended the health span of Ercc1^−/∆^ mice by delaying age-related symptoms and conditions, such as osteoporosis, frailty, and the loss of intervertebral disk proteoglycans [183], and improved the osteogenic capacity of aged bone marrow mesenchymal stem cells both in vitro and in vivo [184]. A recent study revealed that D + Q can improve bone fracture repair in aged mice by removing senescent cells from the callus [185] and decreasing SASP markers [186]. Fisetin, a naturally occurring compound commonly found in many fruits and vegetables, has also been reported to reduce senescent cell burden and its associated inflammation in multiple tissues of progeroid Ercc1^−/Δ^ transgenic mice [187]. Furthermore, when given to wild-type mice at an advanced age, fisetin restored tissue balance, diminished age-related pathologies, and prolonged both median and maximum lifespan [187]. Until now, several classes of senolytics has been developed, including BCL-2 family inhibitors, such as ABT263 [188, 189], ABT737 [190], A13311852 [191], A1155463 [191], and Temozolomide [192]; p53 inhibitors, including UBX0101 [139] and Forkhead box O-4 (FOXO4) D-Retro Inverso (DRI) peptide [40]; and HSP90 inbihitor, 17-DMAG [193]. Some of these senolytics are reported to attenuate bone disorders. For instance, UBX0101, which targets p53, was reported to attenuate the development of post-traumatic OA, reduce pain, and increase cartilage development in aged mice and in p16-3MR transgenic mice [139]. FOXO4-DRI, which disrupts the FOXO4 interaction with p53, was also shown to decrease senescence and its counter features of frailty in fast-aging Xpd^TTD/TTD^ mice [40].

The other class of senotherapeutics is senomorphics, which aim to alleviate the deleterious effects of SASP by reducing inflammation and promoting tissue regeneration without directly eliminating senescent cells. Several approaches to modify SASP include targeting intracellular pro-inflammatory signaling pathways, such as NF-κB, Janus-associated kinase (JAK) inhibitors and AMP-activated protein kinase (AMPK) pathways, and inhibiting mechanistic target of rapamycin (mTOR). Various drugs are used to target NF-kB [194], such as apigenin [195], kaempferol [196], resveratrol [197], and metformin [198]. For example, apigenin has been shown to promote osteogenic differentiation of MSCs, accelerate bone fracture healing by activating the Wnt/β-catenin signaling pathway [199], and prevent bone loss in ovariectomized mice by inhibiting osteoblast and osteoclast differentiation [200]. Kaempferol has also been shown to possess bone-protective properties, such as enhancing osteogenesis and preventing bone loss and fractures in various in vivo [201–205] and in vitro models [206–210] via inhibition of the BMP-2, NF-κB, MAPK, and mTOR signaling pathways [211]. In addition, resveratrol was found to be effective in decreasing SASP factors, such as p16, p21, and p53 through AMPK/ROS signaling, thereby improving osteogenic differentiation [212]. The JAK/STAT pathway is a highly conserved pathway of signal transduction that is involved in immunity, cell proliferation, developmental processes, and more. The JAK/STAT pathway is more highly activated in senescent than non-senescent cells [213]. Blocking the JAK pathway can suppress the SASP in senescent cells, thereby relieving age-associated tissue dysfunction [213–215]. Ruxolitinib, a selective inhibitor of JAK1/2, has been found to reduce systemic inflammation, improve metabolic function, and alleviate frailty in aging mice [216]. It also inhibits progerin-induced senescence in vitro, reduces premature aging phenotypes in Zmpste24-deficient mice [217], decreases SASP expression, and enhances osteogenic differentiation in ovariectomized mice [217]. Tofacitinib, another JAK inhibitor, stabilizes bone density and promotes a positive balance of bone turnover in patients with rheumatoid arthritis [218]. In a recent study, our group used rixolitinib to suppress SASP factors in a glucocorticoid-induced bone-deficit model [132]. Glucocorticoid induced rapid bone marrow adipose (BMAd) senescence. Micro-CT images of distal femur microarchitecture have demonstrated improvement of bone mass when mice were co-treated with synthetic glucocorticoid and ruxolitinib, whereas mice treated with glucocorticoid alone exhibited a low bone mass phenotype [132].

## Conclusion and Perspective

Substantial evidence supports the causal role of cellular senescence in bone tissue during natural aging, premature aging syndromes, and many age-associated skeletal disorders, such as osteoporosis and OA. A central mechanism by which senescent cells expand the senescence program and impair the bone/bone marrow microenvironment is via senescent bone cell–associated SASP, namely “bone-SASP.” It is now well recognized that the SASP is highly heterogeneous, varies depending on cell type and the senescence-inducing stimulus, and is very dynamic, changing over time after the stimulus. Thus, it is important to use a proteomic, unbiased approach to gain insights into highly complex SASP profiles. However, in most studies of the detection of bone-SASP in pathological conditions such as the progeria-associated bone disorders, OA, and osteoporosis, unbiased profiling of the SASP factors was not conducted. Only chosen panels of inflammatory factors and cytokines were detected. Given that the newly generated SenMayo dataset identifies bone-SASP across tissues and species with high fidelity, further detailed characterization and comprehensive identification of the bone-SASP in different age-associated skeletal conditions are warranted. Recent studies suggest that the SASP, as a feature of cellular senescence, not only exerts a detrimental effect locally but may also cause systemic adverse effects. Although the SASP has an endocrine effect on regulating the activities of tissues and organs at remote sites, the endocrine role of the bone-SASP remains largely unexplored. Recent evidence revealed that PDGF-BB produced by senescent preosteoclasts serve as a systemic pro-aging factor that contributes to age-associated increase in arterial stiffness [219] and cerebrovascular impairment [220]. Further assessment is needed of the involvement of bone-derived PDGF-BB in the aging process of other organ systems to validate its endocrine function. Research into the endocrine role of senescent cells is still in the early stage. Given that some bone-SASP factors identified to date are important inflammatory factors and pro-aging factors, there is no doubt that the systemic effect of bone-SASP factors will become one of the main topics in the field of skeletal research. Such research will provide new understanding of the interplay between bone and other organ systems during aging and may yield new strategies to simultaneously treat multiple age-associated disorders.
